# Crystal structure of an aryl cyclo­hexyl nona­noid, an anti­proliferative mol­ecule isolated from the spice *Myristica malabarica*

**DOI:** 10.1107/S2056989016013797

**Published:** 2016-09-05

**Authors:** Ajoy Kumar Bauri, Sabine Foro, Nhu Quynh Nguyen Do

**Affiliations:** a Bio-Organic Division, Bhabha Atomic Research Centre, Trombay, Mumbai 400085, India; b Institute of Materials Science, Darmstadt University of Technology, Alarich-Weiss-Strasse 2, D-64287 Darmstadt, Germany; c Accident and Emergency Department, Franco Vietnamese Hospital, 7 Nguyen Luong Bang Street, Ho Chi Minh City, Vietnam

**Keywords:** crystal structure, aryl cyclo­hexyl nona­noid, *M. malabarica*, anti­proliferative activity, atom disorder and refinement, hydrogen bonding

## Abstract

The title compound, C_21_H_26_O_5_, an aryl cyclo­hexyl nona­noid {systematic name: 3,5-dihy­droxy-2-[9-(4-hy­droxy­phen­yl)nona­noyl]cyclo­hexa-2,4-dien-1-one}, extracted from the spice plant *Myristica malabarica* comprises two ring components, a 4-hy­droxy­phenyl moiety and a 3,5-di­hydroxy­cyclo­hexa-2,4-dienone moiety linked by a nona­noyl chain. The mol­ecule has an extended essentially planar conformation stabilized by an intra­molecular hy­droxy O—H⋯O_carbon­yl_ hydrogen bond, giving a dihedral angle between the two ring systems of 6.37 (15)°. The C, O and H atoms associated with one of the hy­droxy groups of the cyclo­hexa­dienone component are disordered over two sets of sites with site occupancies of 0.6972 and 0.3028. In the crystal, hy­droxy O—H⋯O hydrogen bonds to carbonyl O-atom acceptors form large centrosymmetric *R*_2_^2^(36) cyclic dimers, which are further extended into supra­molecular one-dimensional ribbon structures along [1-11].

## Abstract 2

An aryl cyclo­hexyl nona­noid, an anti­proliferative compound, has been extracted from spice from *M. myristica* using gradient solvent elution. In the crystal, inter­molecular hy­droxy O—H⋯O_carbon­yl_ hydrogen-bonding inter­actions generate large 36-membered centrosymmetric cyclic dimers, which are then extended into one-dimensional ribbons along [1
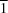
1].

## Chemical context   

The fruit rind of *M. malabarica* (family: *Myristicaceae*) is popularly known as Rampatri in Mumbai, India. It is used as an exotic spice in various Indian cuisines and also as a phytomedicine for the treatment of various kinds of ailments (Forrest & Heacock, 1972 ▸, and references therein). Its major pharmacological activities are credited with hepatoprotective (Morita *et al.*, 2003 ▸), anti-carcinogenic (Patro *et al.*, 2010 ▸; Maity *et al.*, 2012 ▸), anti-leishmanial (Sen *et al.*, 2007 ▸), anti-ulceral (Banerjee *et al.*, 2007 ▸; Banerjee *et al.*, 2008 ▸), anti­proliferative (Manna *et al.*, 2012 ▸, 2015 ▸, 2016 ▸; Tyagi *et al.*, 2014 ▸), anti-inflammatory (Maity *et al.*, 2012 ▸), anti-quorum sensing (Chong *et al.*, 2011 ▸) and anti-thrombotic (Olajide *et al.*, 1999 ▸; Patro *et al.*, 2005 ▸, 2010 ▸) properties and it is found as a constituent in many ayurvedic preparations such as Pasupasi. Previous phytochemical investigations of the fruit rind of *M. malabarica* revealed the presence of four novel diaryl nona­noids named as malabaricones A–D (Purushothaman *et al.*, 1977 ▸) and aryl tetra­deca­noid (Bauri *et al.*, 2016 ▸). In addition, a lignan malabaricanol A and an isoflavone have been isolated from the heart wood of this plant (Purushothaman *et al.*, 1974 ▸; Talukdar *et al.*, 2000 ▸). A detailed phytochemical investigation of a methanol extract of the fruit rind of *M. malabarica* has been carried out. We have isolated a new type of mol­ecule named as an aryl cyclo­hexyl nona­noid, the title compound C_21_H_26_O_5_, as a very minor constituent in addition to the reported compounds malabaricones A–D and aryl tetra­deca­noid. This mol­ecule has exhibited anti-proliferative activity against various cancer cell lines such as A431, U937, MOLT-3, A549 and A2780 by using MTT and western blotting assay (unpublished result). Therefore, based on experimental results, it may be inferred that this fruit rind of *M. malabarica* may be used as a health promoter, a natural remedy which can be prescribed as a botanical dietary supplement to patients who are suffering from these kinds of health problems. The structure of the title compound is reported herein.
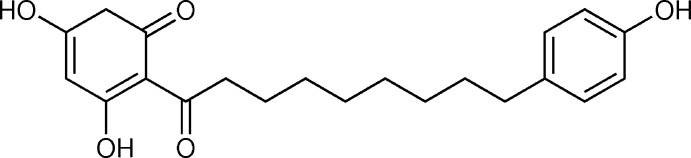


## Structural commentary   

**Figure 1:**
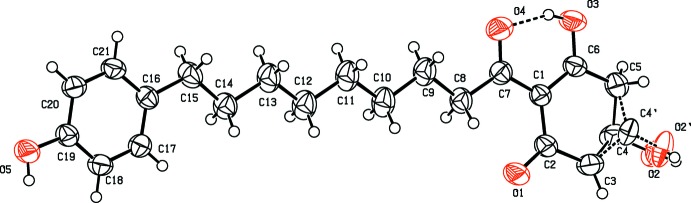
The mol­ecular structure of the title compound, showing the atom labeling and displacement ellipsoids drawn at the 50% probability level. The disordered hy­droxy group (C4—O2—H2*A* and C2′—O2′—H2*B*) is also shown, together with the intra­molecular O—H⋯O hydrogen bond.

The title compound comprises three mol­ecular components, a 4-hy­droxy­phenyl ring, a 3,5-di­hydroxcyclo­hexa-2,5-dienone ring and a bridging nona­noyl moiety (Fig. 1 ▸). The cyclo­hexa­dienone ring has a puckered conformation. There is an intra­molecular O3—H⋯O4_carbon­yl_ bond enclosing an *S*(6) ring motif, which aids in stabilizing the essentially planar overall mol­ecular conformation [inter-ring dihedral angle = 6.37 (15)° and r.m.s. deviation of fitted atoms = 0.2549 Å]. The C, O and H atoms associated with the second hy­droxy group of the cyclo­hexa­dienone component are disordered over two sets of sites (C4, O2, H2*A* and (C4′, O2′, H2*B*) with a site-occupancy factor of 0.6972:0.3028.

## Supra­molecular features   

**Figure 2:**
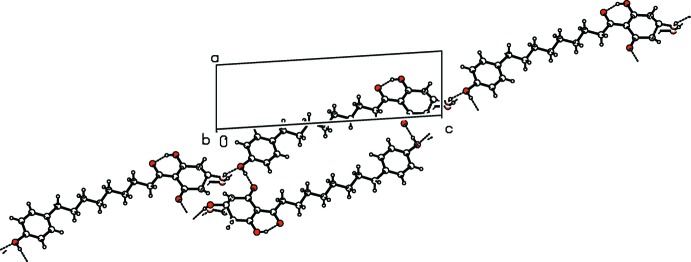
Centrosymmetric dimer formation in the crystal packing of the title compound, with inter­molecular hydrogen bonds shown as dashed lines.

**Figure 3:**
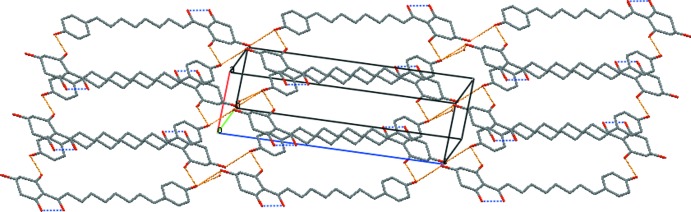
A view of the crystal packing in the unit cell, showing dimer extension into one-dimensional ribbons extending along [1
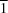
1]. Blue- and orange-coloured dashed lines indicate the intra- and inter­molecular O—H⋯O hydrogen bonding. Only H atoms involved in hydrogen bonds are shown.

In the crystal, the mol­ecules are linked by hy­droxy O5—H⋯O1^ii^ hydrogen bonds to carbonyl O-atom acceptors (Table 1 ▸), forming a primary large centrosymmetric 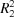
(36) cyclic dimer (Fig. 2 ▸). These dimers are, in turn, linked through the disordered C4 hy­droxy group [O2—H⋯O5^i^ and O2′—H′⋯O5^i^], extending the structure into a one-dimensional ribbon along [1
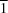
1] (Fig. 3 ▸). No inter-ring π–π inter­actions are present in the structure (minimum ring-centroid separation = 5.66 Å).

## Database survey   

A search of the Cambridge Structural Database (CSD, Version 5.37, updates November, 2015; Groom *et al.*, 2016 ▸) has registered two hits for the compounds found in *M. malabarica*: malabaricone-A (Bauri *et al.*, 2006*a* ▸) and malabaricone-C monohydrate (Bauri *et al.*, 2006*b* ▸), but no other examples were found resembling the title compound.

## Synthesis and crystallization   

The compound has been isolated as a very minor constituent from a methanol extraction of the fruit rind of *M. malabarica* by using CC/SiO_2_ with gradient solvent elution with a binary mixture of solvent methanol and chloro­form. Suitable crystals for X-ray data collection were obtained after recrystallization (×3) from hexa­ne:ethyl acetate (4:1), by slow evaporation at room temperature. The NMR spectroscopic analysis of the crystallized product has been inter­preted as follows. ^1^H NMR data (acetone-*d*_6_, 200 MHz): 8.80 (*s*, *brs*-OH, 1H), 6.89 (*dd*, 1H, *J* = 8.2 Hz, H-2′′ & H-6′′, 2 × Ar-H), 6.59 (*dd*, 2H, *J* = 8.2 Hz, H-3′′ & H-5′′, 2 × Ar-H), 4.20–4.15 (*m*, 1H, H-6), 2.90 (*dd*, 2H, *J* = 7.0 Hz, H-2′), 2.61–2.43 (*dd*, 2H, *J* = 2.20 Hz each, H-4), 2.39 (*dd*, 2H, *J* = 7.0 Hz, H-9) 1.67–1.40 (*m*, 4H, H-3′ & H-8′), 1.19 (*s*, 8H, 4 × –CH_2_ H-4′ H-5′, H-6′ & H-7′). ^13^C NMR data (50 MHz,acetone-*d*_6_): 205.69 (C-1′, >C=O), 198 (C-1, >C=O), 194 (C-3 & C-5, >C=C—OH), 156.20 (C-4′′, Ar—C—OH), 129.94 (C-2′′ & C-6′′, 2 × Ar—C—H), 116.6 (C-3′ & C-5′, Ar—C—H), 134.12 (C-6, >C=C<), 113.60 (C-2, >C=C<), 47.58 (C-2′, –CH_2_—CO–), 42.13 (C-9′, Ar—CH_2_), 40.57 (C-3′, –CH_2_—CH_2_), 35.56 (C-4′, –CH_2_—CH_2_–), 32.37 (C-6′, –CH_2_—CH_2_), 30.19 (C-3′, –CH_2_—CH_2_–), 25.40 (C-5′, –CH_2_).

## Refinement   

Crystal data, data collection and structure refinement details are summarized in Table 2 ▸. The H atoms were positioned with idealized geometry using a riding model with aromatic C—H = 0.93 Å (aromatic) or 0.97 Å (methyl­ene). The H atoms of the OH groups were located in a difference map and were refined as riding on their parent O atoms. All H atoms were refined with isotropic displacement parameters set at 1.2 *U*_eq_ of the parent atom. The atoms C4 and O2 are disordered and were refined using a split model with site-occupancy factors 0.6972:0.3028. The corresponding bond distances in the disordered groups were restrained to be equal. The reflections 0 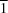 14 and 0 0 7 had poor disagreement with their calculated values and were omitted from the refinement.

## Supplementary Material

Crystal structure: contains datablock(s) I, 1R. DOI: 10.1107/S2056989016013797/zs2367sup1.cif

Structure factors: contains datablock(s) I. DOI: 10.1107/S2056989016013797/zs2367Isup2.hkl

Supporting information file. DOI: 10.1107/S2056989016013797/zs2367Isup3.cml

CCDC reference: 1501296

Additional supporting information: crystallographic information; 3D view; checkCIF report

## Figures and Tables

**Table 1:** Hydrogen-bond geometry (Å, °)

*D*—H⋯*A*	*D*—H	H⋯*A*	*D*⋯*A*	*D*—H⋯*A*
O2—H2*A*⋯O5^i^	0.83	2.18	3.004 (7)	174
O2′—H2*B*⋯O5^i^	0.82	1.86	2.565 (16)	143
O3—H3*O*⋯O4	0.86	1.64	2.440 (3)	153
O5—H5*O*⋯O1^ii^	0.83	1.86	2.687 (3)	172

Symmetry codes: (i) 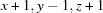; (ii) 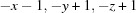.

**Table 2:** Experimental details

Crystal data	
Chemical formula	C_21_H_26_O_5_
*M*_r_	358.42
Crystal system, space group	Triclinic, *P* 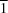
Temperature (K)	293
*a*, *b*, *c* (Å)	5.6630 (8), 8.707 (1), 20.152 (3)
α, β, γ (°)	81.69 (1), 86.48 (1), 88.48 (1)
*V* (Å^3^)	981.2 (2)
*Z*	2
Radiation type	Mo *K*α
μ (mm^−1^)	0.09
Crystal size (mm)	0.48 × 0.48 × 0.20

Data collection	
Diffractometer	Oxford Diffraction Xcalibur diffractometer with Sapphire CCD detector
Absorption correction	Multi-scan (*CrysAlis RED*; Oxford Diffraction, 2009 ▸)
*T*_min_, *T*_max_	0.960, 0.983
No. of measured, independent and observed [*I* > 2σ(*I*)] reflections	6013, 3552, 2638
*R*_int_	0.013
(sin θ/λ)_max_ (Å^−1^)	0.602

Refinement	
*R*[*F* ^2^ > 2σ(*F* ^2^)], *wR*(*F* ^2^), *S*	0.071, 0.178, 1.10
No. of reflections	3552
No. of parameters	254
No. of restraints	3
H-atom treatment	H-atom parameters constrained
Δρ_max_, Δρ_min_ (e Å^−3^)	0.36, −0.20

Computer programs: *CrysAlis CCD* and *CrysAlis RED* (Oxford Diffraction, 2009 ▸), *SHELXS2014* (Sheldrick, 2008 ▸), *SHELXL2014* (Sheldrick, 2015 ▸) and *PLATON* (Spek, 2009 ▸).

## supplementary crystallographic information

### Crystal data

**Table:**

C_21_H_26_O_5_	*Z* = 2
*M**_r_* = 358.42	*F*(000) = 384
Triclinic, *P*1	*D*_x_ = 1.213 Mg m^−^^3^
*a* = 5.6630 (8) Å	Mo *K*α radiation, λ = 0.71073 Å
*b* = 8.707 (1) Å	Cell parameters from 2139 reflections
*c* = 20.152 (3) Å	θ = 2.9–27.7°
α = 81.69 (1)°	µ = 0.09 mm^−^^1^
β = 86.48 (1)°	*T* = 293 K
γ = 88.48 (1)°	Prism, yellow
*V* = 981.2 (2) Å^3^	0.48 × 0.48 × 0.20 mm

### Data collection

**Table:**

Oxford Diffraction Xcalibur diffractometer with Sapphire CCD detector	2638 reflections with *I* > 2σ(*I*)
Radiation source: Enhance (Mo) X-ray source	*R*_int_ = 0.013
Rotation method data acquisition using ω scans	θ_max_ = 25.4°, θ_min_ = 2.9°
Absorption correction: multi-scan (CrysAlis RED; Oxford Diffraction, 2009)	*h* = −6→6
*T*_min_ = 0.960, *T*_max_ = 0.983	*k* = −10→10
6013 measured reflections	*l* = −21→24
3552 independent reflections	

### Refinement

**Table:**

Refinement on *F*^2^	3 restraints
Least-squares matrix: full	Hydrogen site location: mixed
*R*[*F*^2^ > 2σ(*F*^2^)] = 0.071	H-atom parameters constrained
*wR*(*F*^2^) = 0.178	*w* = 1/[σ^2^(*F*_o_^2^) + (0.0517*P*)^2^ + 0.9321*P*] where *P* = (*F*_o_^2^ + 2*F*_c_^2^)/3
*S* = 1.10	(Δ/σ)_max_ < 0.001
3552 reflections	Δρ_max_ = 0.36 e Å^−^^3^
254 parameters	Δρ_min_ = −0.20 e Å^−^^3^

### Special details

**Table:**

Experimental. Absorption correction: CrysAlis RED (Oxford Diffraction, 2009) Empirical absorption correction using spherical harmonics, implemented in SCALE3 ABSPACK scaling algorithm.
Geometry. All esds (except the esd in the dihedral angle between two l.s. planes) are estimated using the full covariance matrix. The cell esds are taken into account individually in the estimation of esds in distances, angles and torsion angles; correlations between esds in cell parameters are only used when they are defined by crystal symmetry. An approximate (isotropic) treatment of cell esds is used for estimating esds involving l.s. planes.

### Fractional atomic coordinates and isotropic or equivalent isotropic displacement parameters (Å^2^)

**Table:**

	*x*	*y*	*z*	*U*_iso_*/*U*_eq_	Occ. (<1)
O1	−0.0508 (4)	−0.0225 (3)	0.83338 (11)	0.0664 (7)	
O2	0.1863 (14)	−0.3525 (8)	1.0267 (3)	0.0655 (19)	0.6972
H2A	0.2365	−0.2936	1.0510	0.079*	0.6972
O2'	0.254 (4)	−0.310 (2)	1.0277 (7)	0.069 (5)	0.3028
H2B	0.2933	−0.2259	1.0380	0.083*	0.3028
O3	0.6549 (4)	−0.3272 (3)	0.82020 (11)	0.0658 (7)	
H3O	0.6550	−0.2796	0.7798	0.079*	
O4	0.5334 (4)	−0.1694 (3)	0.71742 (10)	0.0673 (7)	
O5	−0.5995 (4)	0.8647 (2)	0.10839 (10)	0.0617 (6)	
H5O	−0.7128	0.9054	0.1283	0.074*	
C1	0.3066 (5)	−0.1679 (3)	0.81894 (13)	0.0427 (6)	
C2	0.1065 (5)	−0.1099 (3)	0.85825 (14)	0.0481 (7)	
C3	0.0917 (6)	−0.1598 (4)	0.93293 (15)	0.0742 (11)	
H3	0.0167	−0.0988	0.9625	0.089*	
C4	0.2009 (10)	−0.3083 (6)	0.9562 (2)	0.0636 (14)	0.6972
C4'	0.3050 (18)	−0.2280 (11)	0.9622 (4)	0.046 (2)	0.3028
C5	0.4429 (6)	−0.3327 (4)	0.92481 (15)	0.0605 (9)	
H5A	0.5567	−0.2819	0.9480	0.073*	
H5B	0.4808	−0.4430	0.9313	0.073*	
C6	0.4698 (5)	−0.2731 (3)	0.85166 (14)	0.0470 (7)	
C7	0.3503 (5)	−0.1185 (3)	0.74718 (14)	0.0467 (7)	
C8	0.1815 (5)	−0.0110 (3)	0.70682 (14)	0.0501 (7)	
H8A	0.1566	0.0819	0.7279	0.060*	
H8B	0.0303	−0.0616	0.7084	0.060*	
C9	0.2634 (6)	0.0367 (4)	0.63382 (14)	0.0566 (8)	
H9A	0.3025	−0.0553	0.6131	0.068*	
H9B	0.4049	0.0981	0.6314	0.068*	
C10	0.0701 (6)	0.1314 (4)	0.59556 (15)	0.0598 (8)	
H10A	−0.0672	0.0670	0.5959	0.072*	
H10B	0.0231	0.2186	0.6187	0.072*	
C11	0.1480 (6)	0.1921 (4)	0.52332 (15)	0.0627 (9)	
H11A	0.2042	0.1053	0.5010	0.075*	
H11B	0.2796	0.2612	0.5232	0.075*	
C12	−0.0481 (6)	0.2792 (4)	0.48314 (15)	0.0614 (9)	
H12A	−0.1808	0.2108	0.4836	0.074*	
H12B	−0.1027	0.3672	0.5048	0.074*	
C13	0.0332 (6)	0.3360 (4)	0.41130 (15)	0.0599 (8)	
H13A	0.0865	0.2474	0.3899	0.072*	
H13B	0.1679	0.4026	0.4113	0.072*	
C14	−0.1574 (6)	0.4262 (3)	0.36914 (14)	0.0528 (7)	
H14A	−0.2963	0.3627	0.3705	0.063*	
H14B	−0.2036	0.5197	0.3880	0.063*	
C15	−0.0638 (5)	0.4693 (4)	0.29700 (15)	0.0553 (8)	
H15A	−0.0314	0.3738	0.2783	0.066*	
H15B	0.0863	0.5201	0.2975	0.066*	
C16	−0.2175 (5)	0.5731 (3)	0.24928 (14)	0.0433 (6)	
C17	−0.4281 (5)	0.6465 (3)	0.26883 (14)	0.0486 (7)	
H17	−0.4819	0.6297	0.3138	0.058*	
C18	−0.5602 (5)	0.7439 (3)	0.22330 (14)	0.0492 (7)	
H18	−0.7001	0.7906	0.2376	0.059*	
C19	−0.4795 (5)	0.7697 (3)	0.15660 (14)	0.0458 (7)	
C20	−0.2681 (5)	0.6987 (3)	0.13615 (14)	0.0528 (8)	
H20	−0.2128	0.7169	0.0913	0.063*	
C21	−0.1409 (5)	0.6019 (3)	0.18188 (14)	0.0502 (7)	
H21	−0.0015	0.5550	0.1673	0.060*	

### Atomic displacement parameters (Å^2^)

**Table:**

	*U*^11^	*U*^22^	*U*^33^	*U*^12^	*U*^13^	*U*^23^
O1	0.0639 (14)	0.0742 (15)	0.0563 (13)	0.0366 (12)	−0.0044 (10)	0.0007 (11)
O2	0.082 (5)	0.068 (4)	0.043 (2)	0.013 (3)	0.005 (2)	−0.001 (2)
O2'	0.107 (13)	0.067 (9)	0.032 (5)	−0.021 (6)	−0.013 (5)	0.004 (5)
O3	0.0579 (13)	0.0792 (15)	0.0541 (12)	0.0328 (12)	0.0045 (10)	0.0024 (11)
O4	0.0666 (15)	0.0829 (16)	0.0474 (12)	0.0210 (12)	0.0073 (10)	−0.0015 (11)
O5	0.0625 (14)	0.0668 (14)	0.0496 (12)	0.0272 (11)	−0.0018 (10)	0.0066 (10)
C1	0.0426 (15)	0.0434 (15)	0.0404 (14)	0.0073 (12)	−0.0031 (11)	−0.0018 (11)
C2	0.0477 (16)	0.0482 (16)	0.0463 (16)	0.0125 (13)	−0.0043 (13)	−0.0017 (13)
C3	0.073 (2)	0.099 (3)	0.0426 (17)	0.044 (2)	0.0076 (16)	0.0028 (17)
C4	0.078 (4)	0.070 (3)	0.036 (2)	0.032 (3)	0.010 (2)	0.006 (2)
C4'	0.068 (7)	0.040 (5)	0.030 (5)	−0.002 (5)	−0.007 (4)	−0.005 (4)
C5	0.062 (2)	0.068 (2)	0.0464 (17)	0.0210 (16)	−0.0074 (14)	0.0038 (15)
C6	0.0430 (16)	0.0492 (16)	0.0470 (16)	0.0106 (13)	−0.0005 (12)	−0.0041 (13)
C7	0.0523 (17)	0.0438 (15)	0.0437 (15)	0.0036 (13)	−0.0044 (13)	−0.0053 (12)
C8	0.0565 (18)	0.0486 (16)	0.0429 (15)	0.0030 (14)	−0.0080 (13)	0.0019 (12)
C9	0.071 (2)	0.0545 (18)	0.0433 (16)	0.0027 (15)	−0.0105 (14)	0.0005 (13)
C10	0.078 (2)	0.0532 (18)	0.0467 (17)	0.0009 (16)	−0.0139 (15)	0.0031 (14)
C11	0.081 (2)	0.0580 (19)	0.0471 (17)	0.0033 (17)	−0.0151 (16)	0.0034 (14)
C12	0.081 (2)	0.0522 (18)	0.0492 (17)	0.0053 (16)	−0.0117 (16)	0.0014 (14)
C13	0.074 (2)	0.0544 (18)	0.0488 (17)	0.0063 (16)	−0.0148 (15)	0.0037 (14)
C14	0.0605 (19)	0.0465 (16)	0.0492 (17)	0.0035 (14)	−0.0075 (14)	0.0018 (13)
C15	0.0510 (18)	0.0560 (18)	0.0542 (18)	0.0050 (14)	−0.0073 (14)	0.0082 (14)
C16	0.0428 (15)	0.0400 (14)	0.0455 (15)	0.0013 (12)	−0.0052 (12)	0.0001 (12)
C17	0.0486 (17)	0.0539 (17)	0.0400 (15)	0.0034 (13)	0.0017 (12)	0.0012 (13)
C18	0.0437 (16)	0.0503 (17)	0.0518 (17)	0.0084 (13)	0.0025 (13)	−0.0048 (13)
C19	0.0462 (16)	0.0431 (15)	0.0461 (15)	0.0081 (13)	−0.0040 (12)	−0.0003 (12)
C20	0.0544 (18)	0.0581 (18)	0.0424 (15)	0.0147 (15)	0.0031 (13)	−0.0009 (13)
C21	0.0461 (17)	0.0520 (17)	0.0504 (16)	0.0140 (13)	0.0004 (13)	−0.0039 (13)

### Geometric parameters (Å, º)

**Table:**

O1—C2	1.238 (3)	C10—C11	1.518 (4)
O2—C4	1.415 (7)	C10—H10A	0.9700
O2—H2A	0.8256	C10—H10B	0.9700
O2'—H2B	0.8247	C11—C12	1.534 (4)
O3—C6	1.305 (3)	C11—H11A	0.9700
O3—H3O	0.8588	C11—H11B	0.9700
O4—C7	1.268 (3)	C12—C13	1.509 (4)
O5—C19	1.382 (3)	C12—H12A	0.9700
O5—H5O	0.8342	C12—H12B	0.9700
C1—C6	1.408 (4)	C13—C14	1.543 (4)
C1—C7	1.456 (4)	C13—H13A	0.9700
C1—C2	1.463 (4)	C13—H13B	0.9700
C2—C3	1.503 (4)	C14—C15	1.514 (4)
C3—C4	1.446 (5)	C14—H14A	0.9700
C3—C4'	1.452 (10)	C14—H14B	0.9700
C3—H3	0.9300	C15—C16	1.519 (4)
C4—C5	1.497 (5)	C15—H15A	0.9700
C4'—C5	1.449 (9)	C15—H15B	0.9700
C5—C6	1.490 (4)	C16—C21	1.390 (4)
C5—H5A	0.9700	C16—C17	1.400 (4)
C5—H5B	0.9700	C17—C18	1.395 (4)
C7—C8	1.510 (4)	C17—H17	0.9300
C8—C9	1.517 (4)	C18—C19	1.382 (4)
C8—H8A	0.9700	C18—H18	0.9300
C8—H8B	0.9700	C19—C20	1.400 (4)
C9—C10	1.531 (4)	C20—C21	1.379 (4)
C9—H9A	0.9700	C20—H20	0.9300
C9—H9B	0.9700	C21—H21	0.9300
			
C4—O2—H2A	119.3	C10—C11—C12	113.8 (3)
C6—O3—H3O	105.4	C10—C11—H11A	108.8
C19—O5—H5O	107.1	C12—C11—H11A	108.8
C6—C1—C7	117.7 (2)	C10—C11—H11B	108.8
C6—C1—C2	119.2 (2)	C12—C11—H11B	108.8
C7—C1—C2	123.1 (2)	H11A—C11—H11B	107.7
O1—C2—C1	123.6 (3)	C13—C12—C11	112.6 (3)
O1—C2—C3	118.4 (3)	C13—C12—H12A	109.1
C1—C2—C3	118.0 (2)	C11—C12—H12A	109.1
C4—C3—C2	116.1 (3)	C13—C12—H12B	109.1
C4'—C3—C2	116.5 (4)	C11—C12—H12B	109.1
C4—C3—H3	122.0	H12A—C12—H12B	107.8
C2—C3—H3	122.0	C12—C13—C14	114.5 (3)
O2—C4—C3	115.1 (5)	C12—C13—H13A	108.6
O2—C4—C5	113.0 (5)	C14—C13—H13A	108.6
C3—C4—C5	114.4 (4)	C12—C13—H13B	108.6
C5—C4'—C3	117.1 (6)	C14—C13—H13B	108.6
C4'—C5—C6	112.4 (4)	H13A—C13—H13B	107.6
C6—C5—C4	114.3 (3)	C15—C14—C13	110.4 (3)
C6—C5—H5A	108.7	C15—C14—H14A	109.6
C4—C5—H5A	108.7	C13—C14—H14A	109.6
C6—C5—H5B	108.7	C15—C14—H14B	109.6
C4—C5—H5B	108.7	C13—C14—H14B	109.6
H5A—C5—H5B	107.6	H14A—C14—H14B	108.1
O3—C6—C1	122.7 (3)	C14—C15—C16	118.1 (3)
O3—C6—C5	114.7 (2)	C14—C15—H15A	107.8
C1—C6—C5	122.6 (2)	C16—C15—H15A	107.8
O4—C7—C1	119.1 (2)	C14—C15—H15B	107.8
O4—C7—C8	119.0 (2)	C16—C15—H15B	107.8
C1—C7—C8	122.0 (2)	H15A—C15—H15B	107.1
C7—C8—C9	114.7 (3)	C21—C16—C17	117.4 (2)
C7—C8—H8A	108.6	C21—C16—C15	118.1 (3)
C9—C8—H8A	108.6	C17—C16—C15	124.5 (3)
C7—C8—H8B	108.6	C18—C17—C16	122.4 (3)
C9—C8—H8B	108.6	C18—C17—H17	118.8
H8A—C8—H8B	107.6	C16—C17—H17	118.8
C8—C9—C10	110.7 (3)	C19—C18—C17	118.8 (3)
C8—C9—H9A	109.5	C19—C18—H18	120.6
C10—C9—H9A	109.5	C17—C18—H18	120.6
C8—C9—H9B	109.5	O5—C19—C18	122.4 (2)
C10—C9—H9B	109.5	O5—C19—C20	117.9 (2)
H9A—C9—H9B	108.1	C18—C19—C20	119.7 (2)
C11—C10—C9	113.2 (3)	C21—C20—C19	120.6 (3)
C11—C10—H10A	108.9	C21—C20—H20	119.7
C9—C10—H10A	108.9	C19—C20—H20	119.7
C11—C10—H10B	108.9	C20—C21—C16	121.2 (3)
C9—C10—H10B	108.9	C20—C21—H21	119.4
H10A—C10—H10B	107.8	C16—C21—H21	119.4
			
C6—C1—C2—O1	−176.9 (3)	C6—C1—C7—C8	177.6 (3)
C7—C1—C2—O1	5.0 (5)	C2—C1—C7—C8	−4.3 (4)
C6—C1—C2—C3	3.0 (4)	O4—C7—C8—C9	−5.2 (4)
C7—C1—C2—C3	−175.1 (3)	C1—C7—C8—C9	176.1 (3)
O1—C2—C3—C4	152.2 (4)	C7—C8—C9—C10	174.2 (3)
C1—C2—C3—C4	−27.6 (5)	C8—C9—C10—C11	176.0 (3)
O1—C2—C3—C4'	−164.8 (5)	C9—C10—C11—C12	176.6 (3)
C1—C2—C3—C4'	15.4 (7)	C10—C11—C12—C13	−179.1 (3)
C2—C3—C4—O2	179.6 (5)	C11—C12—C13—C14	−179.2 (3)
C2—C3—C4—C5	46.1 (6)	C12—C13—C14—C15	−176.4 (3)
C2—C3—C4'—C5	−40.0 (10)	C13—C14—C15—C16	−173.8 (3)
C3—C4'—C5—C6	43.6 (9)	C14—C15—C16—C21	−174.6 (3)
O2—C4—C5—C6	−174.4 (5)	C14—C15—C16—C17	7.6 (5)
C3—C4—C5—C6	−39.9 (6)	C21—C16—C17—C18	0.6 (4)
C7—C1—C6—O3	−0.4 (4)	C15—C16—C17—C18	178.4 (3)
C2—C1—C6—O3	−178.6 (3)	C16—C17—C18—C19	−0.4 (5)
C7—C1—C6—C5	−179.6 (3)	C17—C18—C19—O5	−179.9 (3)
C2—C1—C6—C5	2.2 (5)	C17—C18—C19—C20	−0.2 (4)
C4'—C5—C6—O3	155.7 (5)	O5—C19—C20—C21	−179.6 (3)
C4—C5—C6—O3	−163.2 (4)	C18—C19—C20—C21	0.7 (5)
C4'—C5—C6—C1	−25.0 (6)	C19—C20—C21—C16	−0.5 (5)
C4—C5—C6—C1	16.1 (5)	C17—C16—C21—C20	−0.1 (4)
C6—C1—C7—O4	−1.1 (4)	C15—C16—C21—C20	−178.1 (3)
C2—C1—C7—O4	177.0 (3)		

### Hydrogen-bond geometry (Å, º)

**Table:**

*D*—H···*A*	*D*—H	H···*A*	*D*···*A*	*D*—H···*A*
O2—H2*A*···O5^i^	0.83	2.18	3.004 (7)	174
O2′—H2*B*···O5^i^	0.82	1.86	2.565 (16)	143
O3—H3*O*···O4	0.86	1.64	2.440 (3)	153
O5—H5*O*···O1^ii^	0.83	1.86	2.687 (3)	172

Symmetry codes: (i) *x*+1, *y*−1, *z*+1; (ii) −*x*−1, −*y*+1, −*z*+1.
